# A neuronal function of the tumor suppressor protein merlin

**DOI:** 10.1186/s40478-014-0082-1

**Published:** 2014-07-12

**Authors:** Alexander Schulz, Ansgar Zoch, Helen Morrison

**Affiliations:** Leibniz Institute for Age Research, Fritz Lipmann Institute, Beutenbergstrasse 11, D-07745 Jena, Germany; Hans Berger Department of Neurology, Jena University Hospital, Friedrich Schiller University Jena, 07747 Jena, Germany

**Keywords:** Neurofibromatosis type 2, Merlin isoforms, Peripheral neuropathy, Axon-Schwann cell interaction, Tumor development, Intelligence

## Abstract

Mutagenic loss of the *NF2* tumor suppressor gene encoded protein merlin is known to provoke the hereditary neoplasia syndrome, Neurofibromatosis type 2 (NF2). In addition to glial cell-derived tumors in the PNS and CNS, disease-related lesions also affect the skin and the eyes. Furthermore, 60% of NF2 patients suffer from peripheral nerve damage, clinically referred to as peripheral neuropathy. Strikingly, NF2-associated neuropathy often occurs in the absence of nerve damaging tumors, suggesting tumor-independent events. Recent findings indicate an important role of merlin in neuronal cell types concerning neuromorphogenesis, axon structure maintenance and communication between axons and Schwann cells. In this review, we compile clinical and experimental evidences for the underestimated role of the tumor suppressor merlin in the neuronal compartment.

## The hereditary disease Neurofibromatosis type 2

Mutations in the *NF2* gene are causative for the autosomal-dominant disease Neurofibromatosis Type 2 (NF2). This rare multiple neoplasia syndrome affects about 1 in 25,000 live births [1]. However, recent population studies suggest that up to 1 in 300 people will develop a tumor with an underlying sporadic *NF2* mutation during their lifetime [2]. The heritable NF2 disease is mainly characterized by the development of benign Schwann cell-derived tumors, called schwannomas, due to the mutagenic loss of the tumor suppressor merlin. The hallmark feature of NF2 is the bilateral occurrence of schwannomas at the eighth cranial nerve (vestibular schwannoma). These tumors regularly develop in close vicinity to the ‘Obersteiner-Redlich zone’ [3] – the boundary between CNS and PNS – where the transition between Schwann cell and oligodendrocyte myelination takes place. Compressive effects of the schwannoma onto the vestibulo-cochlear nerve may subsequently result in loss of hearing and balance. In addition to vestibular schwannomas and schwannomas occurring within the spinal cord and along peripheral nerves, mutations in the *NF2* gene are responsible for virtually all non-hereditary, sporadically occurring schwannomas and 50% of sporadic meningioma cases [4].

However, NF2 is a clinical syndrome that presents with a variety of other clinical manifestations. In addition to tumors of various entities, NF2 patients suffer from disease-related lesions affecting the skin and the eyes (for detailed review see [5]). Most affected individuals will develop damage to peripheral nerves (peripheral neuropathy) in their lifetime, another common clinical feature in NF2. To date, the pathogenesis of NF2-related neuropathy is not completely understood. Taken together, due to a variety of organ systems being affected by NF2 disease, affected individuals may suffer from severe morbidity in addition to their tumor burden.

Mutations affecting the *NF2* gene may become apparent through at least three kinds of different genetic alterations. Firstly, inherited mutations due to germline mutations result in the loss of one allele; these are accompanied by somatic alterations in the other allele, which cause the hereditary Neurofibromatosis Type 2. Secondly, sporadic schwannomas depend on the acquired somatic mutations in both alleles of the *NF2* gene. Thirdly, as we will discuss later, NF2-related neuropathy may result from the loss of just one allele as a consequence of cell type-specific haploinsufficiency in neuronal cell types.

### Open questions

The tumor suppressor protein merlin, responsible for NF2, is ubiquitously expressed in all tissues during all periods of development [6]. Homozygous deletion of merlin in mice leads to embryonic failure, even before gastrulation [7]. Moreover, conditional ablation of merlin during embryogenesis results in a global tissue fusion defect [8], indicating the importance of merlin from the earliest stage of development. While the role of merlin in glial cell types has been extensively characterized during both development and adulthood, the expression and function in non-tumor related tissues has only occasionally been subjected to mainstream NF2 research.

Microenvironment considerations have become a large field of interest in life science; no given cell type can be comprehensively considered without the context of its environment. Cells in direct or close vicinity influence their neighboring cells - effecting tissue homeostasis. Schwann cells, the origin for NF2-related tumors, are in tight and direct contact with axons-resulting in extensive inter-cellular crosstalk and provoking the hypothesis that axons and/or axon-derived signals, respectively, contribute to tumorigenic activity of Schwann cells. We propose that an exclusive focus on Schwann cell biology in NF2 research risks neglecting not only other high-prevalence symptoms, which occur in NF2 disease, but also potential microenvironmental issues that could contribute to NF2 tumorigenesis. For instance, peripheral neuropathy has been found to appear in individuals who bear mutations in just one merlin allele and lack a significant load of potentially compressive Schwann cell tumors [9]. This led us to the idea that merlin expressed in neurons might have functions unrelated to its tumor suppressor role in glial cells [10].

### The tumor suppressor protein merlin

The human *NF2* gene on Chromosome 22q12.2 comprises 17 exons that encode for the 595 amino acid protein merlin; also known as schwannomin [11,12]. This actin-binding protein belongs to the ezrin–radixin–moesin (ERM) family of proteins that organizes and links membrane proteins to the cortical cytoskeleton [13]. Merlin mediates contact inhibition of proliferation in multiple cell types, including Schwann cells [14] and is reported to target many signaling components to restrict proliferation [15]. For an extensive review of merlin effected pathways please see [16]. Moreover, the tumor suppressor merlin activity is suggested to take place in various cellular compartments, including the cell nucleus [17,18], at the plasma membrane [14,19], in endosomes [20] and even in association with mitotic spindles during mitosis [21]. Although merlin interacts with a high number of different molecules (for detailed review see [22]) in different locations of the cell, we still conclude that part of merlin’s tumor suppressor activity is at the plasma membrane - mediating contact inhibition of proliferation by regulating several small GTPases like Ras or the Rho GTPase family, as well as the Hippo pathway [23].

### Rho GTPases in neuronal cell types

GTPase proteins are molecular switches that regulate many important processes in the cell, including the organization of the actin cytoskeleton [24]. By provoking local actin rearrangements, the protein family of Rho GTPases is essential for the development of highly polarized cells like neurons [25]. Regulators of these small GTPases are therefore of special interest in the broad field of neuromorphogenesis. Merlin has often been shown to exert its various functions through Rho GTPases by determining their activation state [15,26,27]. While GDP-bound molecules are considered to be inactive, GTP-bound proteins actively act on their downstream targets. Considering the significant importance of small GTPases in neuromorphogenesis, merlin, as well as other regulators of small GTPase activity, are plausible candidates for involvement in the vastly complex process of neuronal shape determination. Significantly, mutations in regulators and effectors of Rho GTPases have been associated with diseases of the nervous system, including mental retardation and motor neuron diseases [28].

### The appearance of merlin isoforms

The human gene *NF2* and its close homologue the murine gene *Nf2* are subject to alternative splicing [29]. By far the most abundant isoforms are isoform 1 (595 aa) and isoform 2 (590 aa), which differ in their last 11 and 16 amino acids, respectively [6]. While merlin isoform 1 contains exon 17 instead of exon 16, merlin isoform 2 contains the stop codon bearing exon 16, which results in a C-terminal truncated protein [6].

The altered C-terminus of isoform 2 is hydrophilic and positively charged, while the isoform 1 C-terminus is much less hydrophilic and has no net charge. As a consequence isoform 1 C-terminus binds strongly to the N-terminal FERM domain, while the isoform 2 C-terminus shows only weak binding [30]. Apparently, both C-termini can interact with each other, proposing the formation of isoform hetero-dimers [31]. Due to the structural and charge differences in their very C-terminus, the two main merlin isoforms are likely to have different binding partners in cases where the C-terminus is necessary for protein-protein interactions. So far, only syntenin, an adaptor protein involved in the subcellular trafficking of receptors, has been shown to specifically interact with the C-terminus of isoform 1 [32]. Although merlin has been implicated in receptor trafficking [33], the functional consequence of a specific merlin isoform 1 interaction has not been described.

To date, it remains controversial as to whether both major merlin isoforms exert a tumor suppressive function. No pathogenic mutation that specifically hits one isoform of *NF2* has been described; tumorigenic mutations always inactivate both isoforms [34,35]. The only data on functional differences of the two merlin isoforms comes from *in vitro* studies: Of the two major merlin isoforms, only isoform 1 was originally thought to have proliferation suppressive potential [36,37]. However, more recent studies suggest that both isoforms have equal proliferation inhibiting functions and so far act similarly in most analyzed assays [38–40]. Despite structural differences in the C-terminus, it is reasonable to assume that both isoforms have partially overlapping functions whenever the homologous N-terminus is involved in the regulation of downstream pathways. Furthermore, in an intact cellular system, differences between the two major merlin isoforms may be due to their potential sites of activity - which could be determined by specific isoform binding partners targeting them to distinct cellular and subcellular localizations [41]. Clearly, greater focus and effort is required to identify and catalogue merlin isoforms and their functions. Early studies investigating the spatiotemporal expression pattern of *NF2*/*Nf2* isoforms suggest that merlin plays a pivotal role in neuronal tissue, especially during development. High *NF2* expression was found in brains of humans [42,43] as well as rodents [6,44]. Interestingly, Gutmann et al. reported an increase in isoform 2 expression during neuronal maturation in the cerebral cortex and cerebellum. Additionally, compared to embryonic tissue, neuronal tissue was one of the few organs to retain high expression levels of *Nf2* in adult rats [6]. However, a relevant function of merlin isoform 2, in neurons or other cell types, remained elusive. Only recently have we been able to decipher a unique function for merlin isoform 2; wherein this specific isoform is located and operates in the axonal compartment of neurons [45].

### Expression pattern of merlin in neuronal cells

Although merlin has been studied primarily in glial cells, due to loss of merlin primarily attracting attention by causing benign tumors, several lines of evidence now support additional and functional roles of merlin in neurons. To date, several studies have reported protein expression of merlin in different types of neuronal cells of both the PNS and CNS.

Through different imaging techniques such as immunohistochemistry and in-situ-hybridization, merlin has been detected in sciatic nerve axons [45], in neurons that belong to autonomic ganglia in the intestinal tract [46] and in dorsal root ganglion cells of the PNS [45].

In the CNS, merlin appears in motor neurons of the spinal cord [47], cortical neurons [26,44,47], hippocampal neurons [26,48], neurons of cranial nerve ganglia [47] and in cerebellar Purkinje cells [26,46,47,49]. Particularly in Purkinje cells of the cerebellum, merlin could be functionally associated with neuromorphogenesis and dendritic arborization through the regulation of the small GTPase Rac1 [26]. Furthermore, embryonic expression of merlin in neural stem cells could be demonstrated in neuroepithelial cells of the neural tube, as well as in the ventricular and subventricular zone of the developing brain [8,50]. The analysis of brain tissue and neuronal progenitor cell (NPC) cultures showed consistently that merlin is predominantly present in neurons [51].

On the subcellular level, neuronal merlin was found to be expressed in dendrites [26], in axons [45,52], in the cytoplasm [47,53] and in neuronal synaptic junctions [51]. Conclusively, there is now consistent and broad evidence for a neuronal expression of the tumor suppressor merlin in both rodent and human tissue. However, little is known yet to explain merlin’s function in each cell type. This discrepancy clearly needs to be addressed in the future.

### Polyneuropathy in NF2 patients

Besides the development of multiple gliogenic tumors affecting both the PNS and CNS, many NF2 patients will develop peripheral neuropathy during their lifetime. Affected individuals can suffer from stocking-like hypoalgesia (reduced sensitivity to pain) and hypesthesia (decreased tactile sensibility) as well as loss of vibration sense (pallhypesthesia). Patients may also present with a distal reflex loss that can be followed by a slow but progressive distal muscle atrophy and paresis in later stages of the disease [54]. Usually, peripheral neuropathy can occur as a rather local phenomenon (mononeuropathy simplex or multiplex) or a more generalized event (polyneuropathy).

Indeed, the exact proportion of NF2 sufferers who develop peripheral nerve damage remains obscure, as prevalence numbers vary largely. In a huge clinical study, peripheral nerve lesions unrelated to tumor masses were observed in 6% of patients suffering from NF2 [55]. Another investigation, with primary focus on NF2-related neuropathy, found that clinical signs manifesting as peripheral neuropathy occurred in 47% of investigated patients [56]. Further electrophysiological examination even revealed evidence of neuropathy in 67% of those individuals.

The general observation that many NF2 patients present with areflexia, which cannot be completely explained by the actual tumor load, suggests that subclinical or masked neuropathy is potentially underdiagnosed in NF2 disease [57].

### Electrophysiological methods as a diagnostic tool for neuromuscular diseases

Electrophysiological measurements are an indispensable tool for investigating the functional integrity of peripheral nerves in both the clinical and laboratory environment [58]. In humans, a large number of neuromuscular disorders and neuropathies diagnostically rely on electrophysiological measurements. By measuring nerve properties as conduction velocity or potential amplitudes of the signal, it is possible to characterize the origin of peripheral nerve diseases. The nerve conduction velocity is highly dependent on rapid signal propagation enabled by myelination. Therefore, demyelinating processes generally show decreased conduction velocities. When significantly reduced, the compound motor action potential (CMAP) – correlating with the number of functional axons – is an indicator for axonal damage. Hence, by means of electrophysiological methods, the etiology of peripheral nerve damage can be discriminated; such as for hereditary neuropathies, diabetic neuropathy, chronic inflammatory demyelinating polyneuropathies (CIDP) or metabolic neuropathies.

### Exploring the pathogenesis of NF2-related neuropathy

Originally, schwannomas were held to primarily account for observable neuropathic symptoms developing in the course of NF2 [59]. Schwannomas can occur within the spinal cord, on spinal nerve roots, along peripheral nerves and around cranial nerves – with the vestibular nerve being the most frequently involved cranial nerve. The localization of a given tumor naturally determines the presenting features and symptoms of an individual, e.g. affections of a spinal nerve root by a tumor may cause motoric and sensory problems that are clearly related to its innervation area. However, for the most part, clinical signs of neuropathy appear independently from the site of peripheral nerve schwannomas. In single NF2 patients, polyneuropathy even developed years before other NF2-related symptoms, like tumors, became evident [56].

Although benign in nature, schwannomas are thought to produce pain and other symptoms by compressive effects, thereby impairing axonal integrity in a given nerve. However, the clinical appearance of neuropathy can hardly be explained by the tumor burden alone. Concretely, in some NF2 patients suffering from polyneuropathy, muscle weakness occurs without significant spinal or peripheral nerve tumor burden, suggesting that factors other than gross tumor growth might be responsible for this disorder [60]. Besides, surgical resection of gross tumor load along peripheral nerves often lacks a beneficial outcome for affected individuals in terms of neuropathic symptoms [61]. Furthermore, NF2-associated polyneuropathy typically involves more than two peripheral nerves and predominantly affects extremities in a distal and symmetric fashion [56,62,63], suggesting a systemic rather than local issue. Thus, tumorlets - hyperproliferative Schwann cells – are also unlikely to explain the complete etiology of peripheral neuropathy in these patients [9]. However, a high-resolution MRI study – aiming to link tumor load with severity of polyneuropathy in NF2 patients – indicated that non-compressive fascicular microlesions along peripheral nerves, correlated with severity of clinical symptoms of NF2-related neuropathy. Apart from that, compressive tumor macrolesions were absent in most neuropathy-affected extremities [61].

Both neuropathological and electrophysiological investigations initially suggested that NF2-related polyneuropathy might develop independently of large solitary schwannomas [64]. Hagel et al. provided evidence for an axon-intrinsic pathogenesis of neuropathy in sural nerve biopsies indicated by pathological reduction of nerve fiber densities, accompanied by diffuse proliferation of Schwann cells. Onion bulbs, pathological indicators of repetitive de-/remyelination, were just seen in a subset of investigated patients [60]. Furthermore, by determining nerve conduction properties, two studies were able to show that NF2-related peripheral neuropathy is commonly of axonal origin [56,61]. In the majority of cases, the nerve conduction velocity appeared normal (above the reference levels of the tested nerves), while CMAP values were markedly decreased – a diagnostic combination suggestive of axonal neuropathy.

**Figure 1:**
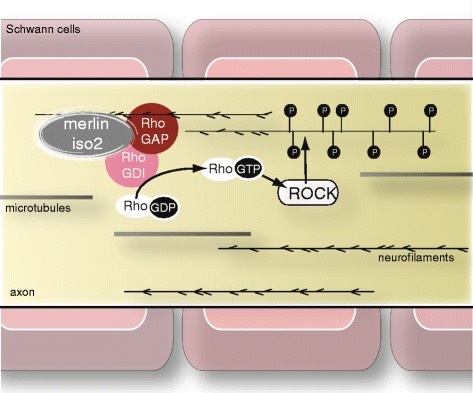
**Potential role for merlin isoform 2 in NF2-related neuropathy.** Merlin isoform 2 in axons assembles a multi-protein complex with RhoGDI [68] and RhoGAP that leads to the local activation of the small GTPase RhoA by GTP loading [45]. This results in subsequent neurofilament phosphorylation through Rho-associated kinase (ROCK). The specific loss of merlin isoform 2 can therefore provoke irregular neurofilament phosphorylation and impaired axon structure maintenance.

It was previously hypothesized that NF2 and axonal neuropathies would exist as independent diseases [65,66]. Our group recently deciphered a promising pathomechanism indicating how the loss of merlin could contribute to the development of NF2-related neuropathy in an axon-intrinsic manner [45]. Specifically, via the GTPase Rho/RhoKinase signaling network, merlin’s splice variant isoform 2 promotes phosphorylation of neurofilaments that are neuron-specific intermediate filaments essential for axon structure and caliber [67]. Using a mouse model bearing loss of merlin isoform 2, as well as sural nerve biopsies of NF2 patients, we could show that proper merlin signaling in axons (see Figure 1) is essential for axon structure maintenance [45]. Strikingly, heterozygous deletion of *Nf2* isoform 2 caused haploinsufficiency *in vivo*. This is consistent with clinical findings that *NF2* germline mutations are sufficient to cause polyneuropathy; the loss of the second allele is not required in humans [9].

### Merlin in axon-Schwann cell interactions

The implication of merlin in prevention of Schwann cell tumorigenesis has been extensively studied [10,69]. NF2-related schwannomas are encapsulated tumors composed almost entirely of Schwann cells perched on, but not commingled with, normal nerve bundles [70]. However, the benign dignity of NF2-associated Schwann cell-derived tumors is accompanied by sparse response to classical chemotherapy [71].

Importantly, the role of merlin in Schwann cells is not just restricted to its tumor suppressive function. It has been reported to play a critical role in the control of Schwann cell numbers and is necessary for the correct organization and regulation of axo-glial heterotypic contacts [72]. Consistently, merlin in Schwann cells has been reported to promote their alignment along axons and ultimately influences myelin segment length [73].

Generally, the behavior of Schwann cells is strictly under the control of axonal signals, both during development and in adulthood [74]. As such, Schwann cell actions should not be assessed solely by endogenous Schwann cell signaling pathways, but rather with respect to the influence of axons and vice versa. Signals from axons regulate the intimate communication of Schwann cells with axons of the PNS, provide proliferative and survival signals, and determine the polarization and differentiation programs to either non-myelinating or myelinating phenotypes [75,76]. Moreover, axonal damage triggers rapid Schwann cell de-differentiation and this is accompanied by myelin breakdown, Schwann cell detachment from axons and subsequent proliferation [77]. Typically, patients with NF2 present with different types of benign Schwann cell tumors, in which most Schwann cells lose contact with axons [70,78]. Focusing on the pathogenesis of polyneuropathy affecting NF2 patients, Sperfeld and colleagues [56] also suggested that the nerve damaging disease could possibly occur because Schwann cells can no longer adhere properly to the axons. This underlines the importance of the microenvironment of peripheral nerves, where damage to one cell type invariably leads to pathophysiological changes in the other [79].

The literature contains several reported observations suggesting that neuronally expressed merlin could also be involved in the tightly regulated crosstalk between axonal processes and Schwann cells. For instance, N-terminal merlin can be associated with Caspr/paranodin, an axonal transmembrane glycoprotein enriched at paranodal junctions and important for the reciprocal axo-glial signaling [80]. Merlin also interacts with βII-spectrin - another molecule supporting the axonal cytoskeleton at paranodes (see Table 1) - essential for myelinated axon domain organization [30,49,81]. Paranodal junctions, in general, are specialized molecular domains of myelinated axons that are thought to promote adhesion between Schwann cells and axons (for detailed review see [82]). This data implies that neuronally expressed merlin could be directly involved in the mechanism determining proper axon-Schwann cell contact formation.

**Table 1:** **Binding or interaction partners of neuronal merlin**

**Protein**	**Cell type; species**	**Reference**
Neurofilaments	DRG, sciatic nerve lysates (mouse)	[45]
Riβ (PKA subunit)	brain lysates (rat)	[83]
βII-spectrin	Purkinje cells	[30,49]
Caspr/paranodin	brain extracts (rat)	[80]
Paxillin	neuroblastoma cells (mouse)	[84]
RhoGDI	Cell lysate from primary neurons (mouse)	[45,68]
p190RhoGap	Sciatic nerve lysates (mouse)	[45]

**Figure 2:**
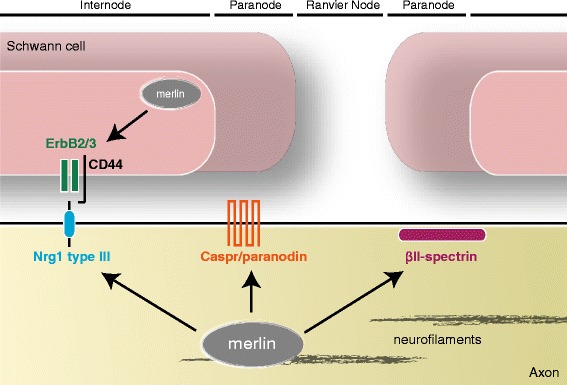
**Interaction of neuronally expressed merlin with axonal proteins essential for axon-Schwann cell signaling.** Merlin in neurons has been shown to interact with two axonal proteins in the paranode region of myelinated axons: Caspr/paranodin [80] and βII-spectrin [30]. Furthermore, merlin regulates the expression of Nrg1 type III [85], an axon surface molecule with growth factor-like impact on Schwann cell behavior. Interestingly, the receptor of Nrg1 type III on Schwann cells, ErBB2/3 [33] as well as its co-receptor CD44 [14], is regulated by merlin expressed in Schwann cells.

Recently, we analyzed the impact of neuronally expressed merlin on the best-characterized signaling cascade between axon and Schwann cells, namely the Neuregulin1 - ErbB2/3 pathway [85]. We were intrigued to find that the Neuregulin splice variant Nrg1 type III, expressed on axonal membranes as a juxtacrine growth factor molecule for Schwann cells, shows reduced expression following loss of merlin *in vitro* and *in vivo* (see Figure 2). In contrast to merlin isoform 2, which is specifically implicated in axon structure maintenance, both major merlin isoforms appear to have equal potency in affecting Nrg1 type III.

In accordance, human sural nerve biopsies taken from NF2 patients suffering from polyneuropathy display a strong and consistent reduction of Nrg1 type III. This is accompanied by a compensatory up-regulation of ErbB2 expression on Schwann cells; as analyzed in mice bearing neuron-specific merlin knockout as well as NF2 patient samples. Notably, the expression abnormalities of both Nrg1 type III and ErbB2 receptor appear to be very specific to NF2 disease and much more pronounced than in other axonal types of neuropathies [85].

ErbB2/ErbB3 heterodimers are neuregulin receptors which are required for SC development [86]. In mature peripheral nerves, Nrg1 participates in the regeneration and re-myelination of injured myelinated fibers, processes that involve SC de-differentiation, proliferation and re-differentiation to a myelinating phenotype [87]. Interestingly, ErbB2/3 receptor overexpression has been linked to the pathogenesis of one type of demyelinating neuropathy occurring in the course of Charcot-Marie-Tooth disease type 1 [88], which raises the possibility of Schwann cell-autonomous effects on the development of neuropathies.

Remarkably, the loss of merlin in primary Schwann cells is associated with elevated levels of ErbB receptors [33]. Furthermore, merlin in Schwann cells interacts with CD44 [14], a membrane glycoprotein that enhances neuregulin-induced ErbB2 phosphorylation [89]. Concerning the regulation of ErbB2/3 receptor expression, merlin obviously has synergistic functions in neurons and Schwann cells, arguing for a holistic function of merlin in both cellular compartments of peripheral nerves. Consequently, ErbB2/3 receptor overexpression has been identified as a potential target for NF2 therapy [90], using the monoclonal antibody Trastuzumab [91] or the tyrosine kinase inhibitor Lapatinib [92].

Axons are thought to maintain Schwann cells in a differentiated state during adulthood, to ensure the correct functioning of the nerve [75,76]. It is therefore reasonable to assume that misregulation of axon surface proteins–essential for Schwann cell alignment and differentiation – could contribute to the initial events in tumor development. Notably, a comparable pathogenesis is likely to occur in the related tumor syndrome Neurofibromatosis Type 1 (NF1); possibly the most common inherited disorder caused by a single gene, which is characterized by the development of multiple neurofibromas. Since heterotypic cell–cell contacts control cell proliferation and suppress tumorigenesis [93], the loss of Schwann cell contact to axons is a frequent and important early event in tumor development for these highly heterotypic benign tumors of the peripheral nerve sheath. Joseph et al. [94] could additionally show that NF1-related tumors arise from differentiated glial cells instead of undifferentiated neural crest cells. These results further support the hypothesis that Schwann cell detachment from axons is an important early event in tumor development – a mechanism neuronally expressed merlin could be also involved in concerning NF2 disease. Taken together, new findings on merlin in the neuronal compartment suggest pathogenesis of NF2 disease, wherein the *NF2* gene encoded protein has cell type-dependent functions in order to prevent tumor formation.

### Concluding remarks

In a recent study [45] the impressive number of proteins merlin can interact with was further expanded (for detailed review see [22]). More precisely, merlin has now been reported to associate with all three classes of cytoskeletal elements; namely actin filaments [95], microtubules [21,96] and intermediate filaments [45]. This connection underlines merlin’s fascinating role as a versatile cytoskeleton associated molecule involved in a vast variety of signaling events. This makes merlin a highly enigmatic and extraordinary tumor suppressor. However, the contribution of merlin splice variants may play an additional role for the interaction with multiple proteins in a large variety of different cell types.

According to Knudson’s two-hit-hypothesis, merlin acts as a classical tumor suppressor. Interestingly, inactivating mutations of merlin seem to have tissue-dependent diverse effects. Loss of heterozygosity, the functional loss of one gene allele in which the other allele was already inactivated, is known to be crucial for merlin owed tumor formation in Schwann cells [97,98]. However, deficient effects due to loss of merlin can already be detected in neurons where only one mutation is verifiable [9]; thus explaining why polyneuropathy in NF2 patients is frequently found in the absence of compressive tumors and may even appear chronologically earlier. In line with this notion, axons of mice heterozygous for merlin isoform 2 mutations show functional and morphological abnormalities [45]. However, despite the clear relevance of merlin in neuronal cells of the PNS, a functional role for merlin in CNS neurons remains elusive.

Grönholm et al. [83] provided a first functional hint for neuronally expressed merlin in the CNS. It was shown to be the first known binding partner of Riβ, a regulatory subunit of protein kinase A (PKA), which is evidently implicated in learning-related functions [99]. Consistently, Wassink and colleagues [100] reported that merlin is a candidate gene for the development of autism spectrum disorder (ASD), which has been shown to be associated with dendritic spine abnormalities [101]. Because dendritic spine morphology is in turn highly susceptible to the activation state of small GTPases [102], an impact of merlin on spine morphology and/or plasticity is very likely but, as yet, defies characterization. In line with this hypothesis, the loss of merlin in neural progenitor cells results in severe reduction in hippocampus size [50]; the implications of which in learning and memory acquisition are indisputable. However, despite merlin’s theoretical implication in learning and memory acquisition, no study has ever suggested changes in NF2 patients’ intelligence or cognitive performance. If such potential effects on learning and memory were to exist, these could be rather subtle and/or hidden by the vast environmental noise that envelops human intelligence.

### Outstanding questions

- How can future disease models and considerations regarding NF2 pathogenesis better emphasize the importance of the nerve microenvironment?
- Does the loss of neuronal merlin and its influence on Schwann cell behavior impair peripheral nerve regeneration following injury?
- Does merlin deficiency in neurons contribute to NF2-related schwannoma formation?
- Is the downstream signaling of axonal merlin isoform 2 – involving RhoA and ROCK – relevant for other hereditary neuropathies whose mechanisms have yet to be deciphered?
- Are Schwann cell-autonomous effects of merlin due to loss-of-heterozygosity sufficient to promote NF2-related neuropathy without any disturbances in the axonal compartment?
- Are there alterations in cognitive performances in merlin-deficient animals and patients suffering from the NF2 disease?
- What are the specific functions of the two major merlin isoforms? Is there a cell type-specific expression? With regard to the variety of different merlin functions, which merlin isoforms can compensate for each other?
